# Prefrontal cortex activation by binge-eating status in individuals with obesity while attempting to reappraise responses to food using functional near infrared spectroscopy

**DOI:** 10.1007/s40519-023-01558-z

**Published:** 2023-03-30

**Authors:** Megan N. Parker, Helen Burton Murray, Amani D. Piers, Alexandra Muratore, Michael R. Lowe, Stephanie M. Manasse, Hasan Ayaz, Adrienne S. Juarascio

**Affiliations:** 1 Department of Psychology, Drexel University, Philadelphia, PA USA; 2 The WELL Center, Drexel University, Philadelphia, PA USA; 3 Department of Medical and Clinical Psychology, Uniformed Services University of the Health Sciences (USUHS), Bethesda, MD USA; 4 Section on Growth and Obesity, Division of Intramural Research, Eunice Kennedy Shriver National Institute of Child Health and Human Development (NICHD), National Institutes of Health (NIH), Bethesda, MD USA; 5 Department of Medicine, Division of Gastroenterology, Massachusetts General Hospital, 55 Fruit Street, Boston, MA 02114 USA; 6 Harvard Medical School, Boston, MA USA; 7 School of Biomedical Engineering, Science and Health Systems, Drexel University, Philadelphia, PA USA; 8 Drexel Solutions Institute, Drexel University, Philadelphia, PA USA; 9 Department of Family and Community Health, University of Pennsylvania, Philadelphia, PA USA; 10 Center for Injury Research and Prevention, Children’s Hospital of Philadelphia, Philadelphia, PA USA

**Keywords:** Binge eating, Obesity, Inhibitory control, Inhibition, Reappraisal, Neuroimaging

## Abstract

**Purpose:**

Difficulty reappraising drives to consume palatable foods may promote poorer inhibition and binge eating (BE) in adults with obesity, but neural underpinnings of food-related reappraisal are underexamined.

**Methods:**

To examine neural correlates of food-related reappraisal, adults with obesity with and without BE wore a portable neuroimaging tool, functional near-infrared spectroscopy (fNIRS). fNIRS measured activity in the prefrontal cortex while participants watched videos of food and attempt to “resist” the food stimuli (i.e., “consider the negative consequences of eating the food”).

**Results:**

Participants (N = 32, 62.5% female; BMI 38.6 $\pm$ 7.1; 43.5 $\pm$ 13.4 y) had a BMI > 30 kg/m^2^. Eighteen adults (67.0% female; BMI 38.2 $\pm$ 7.6) reported BE (≥ 12 BE-episodes in preceding 3 months). The control group comprised 14 adults who denied BE (64.0% female; BMI 39.2 $\pm$ 6.6). Among the entire sample, mixed models showed significant, small hyperactivation during crave and resist compared to watch (relax) condition bilaterally in the medial superior frontal gyrus, dorsolateral areas, and middle frontal gyrus (optodes 5, 7, 9, 10, 11, and 12) in the total sample. No statistically significant differences in neural activation were observed between the BE and control group. Moreover, there were no significant group by condition interactions on neural activation.

**Conclusion:**

Among adults with obesity, BE status was not linked to differential activation in inhibitory prefrontal cortex areas during a food-related reappraisal task. Future research is needed with larger samples, adults without obesity, and inhibition paradigms with both behavioral and cognitive components.

**Level of evidence:**

Level III: Evidence obtained from well-designed cohort or case–control analytic studies.

*Trial registration*: # NCT03113669, date April 13, 2017.

**Supplementary Information:**

The online version contains supplementary material available at 10.1007/s40519-023-01558-z.

## Introduction

Binge eating (BE) is characterized by a sense of loss of control while consuming unambiguously large amounts of food and is reported by 9–29% of adults with obesity [15]. Dysregulation in neurocognitive processes such as an imbalance between increased reward response to food and decreased food-related proactive inhibition (e.g., having a thought that one should eat food and not acting on it) may promote BE among adults with high weight [17]. Individuals with BE have difficulty resisting drives to consume food or considering the potential longer-term negative consequences of BE [17]. Given deficits in proactive inhibition, engagement in food-based cognitive reappraisal (i.e., cognitively reassessing one’s initial reaction to a food) may help adults increase control over drives to eat, thereby strengthening capacity for proactive inhibition. Food and non-food-based inhibition deficits have been linked to hypoactivation in dorsal and orbitofrontal areas of the prefrontal cortex (PFC) [9, 16]. However, it is unknown whether, among individuals with obesity, activation in regions of the brain associated with inhibition and reappraisal is linked to BE. Thus, elucidation of neural correlates of decreased food-related reappraisal could inform neural signatures of BE in adults with obesity.

Functional near-infrared spectroscopy (fNIRS) is an increasingly used neuroimaging method for brain function research and has been recommended for use among individuals with obesity and eating disorders [18]. fNIRS is a wearable device used to assess neural activity by measuring changes in cortical hemodynamic response with centimeter level spatial resolution. fNIRS offers multiple advantages over MRI including relatively low maintenance costs, less technical expertise required, temporal resolution superior to that of fMRI, and ability to study individuals with metal in their body. Perhaps most relevant for research on weight and eating, fNIRS does not require a constrained environment, so individuals with larger bodies, who may feel uncomfortable or claustrophobic in compact MRI machines, can comfortably complete an assessment with fNIRS.

This study aimed to use fNIRS to measure neural activation during a cognitive, food-based reappraisal task that is designed to evoke activation in areas of the brain related to food-related proactive inhibition. Weight status may independently contribute to inhibitory control differences [12], thus we aimed to compare neural activation in adults with obesity and BE compared to adults with obesity but no BE. Consistent with prior research [4, 9], we hypothesized that BE status would be linked to lower activation in PFC areas related to inhibition and appraisal (e.g., dorsal and orbitofrontal) when attempting to cognitively resist the temptation to consume food.

## Methods

### Participants and procedures

Participants with BE were recruited for a study utilizing guided self-help cognitive–behavioral therapy for BE and completed all procedures presented in the current study prior to starting therapy. Individuals with ≥ 12 binge-eating episodes (subjective or objective in size), were recruited as previous findings have suggested that the size of BE-episodes does not necessarily correlate with illness severity or inhibition [14]. A control group of adults with obesity without BE, were recruited for a one-time research assessment. Participants in both the BE and control group were eligible if they were fluent in English, 18 years or older, and had a BMI of 30 kg/m^2^ or greater. Participants were excluded if they were engaging in compensatory behaviors (e.g., self-induced vomiting, laxative/diuretic use, excessive and driven exercise), receiving eating disorder or weight loss treatment, currently using a stimulant medication, or reported any of the following during a phone screening with a trained assessor: acute suicide risk, a current co-morbid psychological disorder that required attention beyond the study (e.g., psychotic disorder, substance dependence), intellectual disability or autism spectrum disorder, history of a neurological condition or traumatic brain injury, current pregnancy, or past bariatric surgery. Additionally, participants in the control group were excluded if they had a history of any eating disorder diagnosis, reported past or current binge-eating episodes (subjective or objective in size), or had a history of a clinically significant psychological disorder (e.g., psychotic disorder, substance dependence).

This study was approved by the Drexel University Institutional Review Board. Participants provided written informed consent and completed an in-person study visit. Participants were asked to eat a meal (i.e., a typical full-sized meal for the participant) two hours prior to the visit then fast until the visit. During the visit, participants provided ratings of hunger and fullness, underwent a fNIRS scan, completed self-report questionnaires, and were administered a clinical interview to confirm presence or absence of BE.

## Measures

### Eating disorder pathology

The Eating Disorder Examination (EDE; [7] is a widely used and validated, rater-administered measure for BE. The frequency of BE-episodes and compensatory behaviors in the prior three months was assessed. Participants also reported on the amount of food consumed during BE-episodes, and episodes were categorized as objectively or subjectively large according to the EDE guidelines. The EDE also provides a measure of dietary restraint (restraint subscale) and global eating disorder symptom severity.

### fNIRS

Prefrontal cortex activation was measured by fNIRS, a portable, headband-like optical neuroimaging device. fNIRS has optodes that  represent channels or measurement areas akin to fMRI “voxels” and measures continuous changes in cortical oxygenation via near-infrared light [10]. fNIRS passes near-infrared light through the scalp and measures levels of reflected light to operationalize neural activation by changes in oxygenated and deoxygenated hemoglobin. We used a modified Beer–Lambert law with 2 Hz sampling rate with dual wavelength (730 nm and 850 nm) and ambient channels (for signal quality assessment) for each measurement location. The fNIRS sensor pad has up to 16 optodes with 2.5 cm source-detector distance organized in a 2 by 8 rectangular grid [2]; however, the sensor used in this study had 3 disconnected and hence unavailable optodes in the right ventrolateral region (see Additional file 1: Fig. S1). Source-detector separation was approximately 2.5 cm. A trained technician cleaned participants’ foreheads using an alcohol swab prior to sensor probe placement on the forehead. We used nasion and inion anatomical landmarks to standardize sensor placement as described before [3].

### Cognitive reappraisal task

While wearing the fNIRS sensor, participants completed a food version of the computerized Crave–Resist task [11] presented using E-Prime software. The task (see Additional file 1: Fig. S2) prompted participants to watch videos of food (e.g., hands dipping french fries in ketchup) and either to Crave (“Focus on the enjoyment of consuming the food, the flavors and sensations of eating it”) or to Resist (“Consider the possible negative consequences of over-consuming the food; try to resist or suppress the temptation to consume the food”). Non-food images were shown during the control, or Watch, condition. Specifically, participants are instructed to simply watch the non-food videos (e.g., hands typing on a keyboard). All videos lasted 5 s. Participants completed six trials, each containing one Crave, Resist, and Watch video. Instruction order was randomized across the trials.

The videos were cropped and adjusted for visual components (brightness, video quality) to match across conditions. For a subset of participants (*n* = 9 BE; *n* = 6 control), immediately after completing the task, the assessor asked the participants to rate perceived success engaging with the “Crave” and “Resist” conditions. Assessors asked participants: “For the times you were asked to Crave, on a scale of 0–10 how successful do you think you were—with 0 being unsuccessful and 10 being very successful?” The same question was asked for the Resist condition.

### Covariates

Participants self-reported demographics. Participants’ body weight (on a calibrated scale) and height (on a stadiometer) was measured in triplicate and BMI was calculated from average measurements. The Beck Depression Inventory-II [6] includes 21 items, with higher scores indicating greater depression symptom severity over the past 2 weeks; Cronbach alpha in the current sample was 0.90. Prior to completing fNIRS, participants responded on a visual analogue scale—“How hungry do you feel?” (0 = “I am not hungry at all”; 100 = “I have never been more hungry”)” and “How strong is your desire to eat your favorite food?” (0 = “Not strong at all”; 100 = “Extremely strong”).

### Statistical analysis

Raw fNIRS light intensities were pre-processed for signal quality. High-frequency noise and physiological artifacts (e.g., heart rate, respiration rate, cardiac cycle effects) were removed by using a low-pass filter with a finite impulse response filter and a cutoff frequency of 0.1 Hz [10]. Using sliding-window motion artifact rejection [1], light intensity values were scanned for artifacts from motion or poor sensor–scalp coupling and confirmed with visual inspection. Prefrontal activation was calculated by averaging changes in localized oxyhemoglobin and deoxyhemoglobin concentrations for each of 13 optodes, separately, using the Modified Beer–Lambert law for task periods. Oxygenated hemoglobin concentrations have a stronger relation with fMRI than deoxygenated hemoglobin. Thus, changes in oxygenated hemoglobin are reported for main analyses. Deoxygenated hemoglobin results are reported in Supplementary Table 1. Optode/channel-based analysis were conducted. Findings are reported both by individual optode number, and hypothesized corresponding brain regions as identified by prior research on optode–brain region relations [13].

Linear mixed models with repeated measures were used to test for differences in brain activation during the Crave–Resist task between BE and control groups. Models were estimated in NCSS analysis software. A restricted maximum likelihood, and diagonal variance–covariance matrix were utilized. Performance across task conditions were nested within participant. Fixed factors were task condition and group (BE/control). Given that this was a pilot study, effect sizes, rather than p-values are used to interpret results. Partial eta-squared ($\eta_{p}^{2}$) was used as a measure of between subject effect sizes and was interpreted as small ($\eta_{p}^{2}$ = 0.01), medium ($\eta_{p}^{2}$ = 0.06), or large ($\eta_{p}^{2}$ = 0.14). Compared to the control group, the BE group had significantly higher ratings of depressive symptoms, and dietary restraint; analyses re-run adjusting for these factors are reported in the Additional file 1.

## Results

### Group characteristics

Thirty-seven participants were recruited. Thirty-two adults (62.5% female; BMI 38.6 $\pm$ 7.1; 43.5 $\pm$ 13.4 y, 18 with BE and 14 without BE) provided usable data. Missing data are reported in the Additional file 1. Group demographics and baseline characteristics are reported in Table 1. Groups did not significantly differ on age, BMI, sex, or race.

**Table 1:** Characteristics of adults with and without binge eating

	Total sample (*n* = 32)	Binge eating group (*n* = 18)	Obesity control group (*n* = 14)	Group comparisons	Effect size
		*n, %*	*n, %*	X^2^*, p*	
Sex (female)	20, 62.5%	12, 66.7%	9, 64.3%	0.1, 0.89	–
Race^a^				3.8, 0.29	–
Asian	1, 3.1%	1, 5.6%	0, 0.0%	–	–
Black	9, 28.1%	4, 22.2%	5, 35.7%	–	–
Other	1, 3.1%	0, 0.0%	1, 7.1%	–	–
white	21, 65.6%	13, 72.2%	8, 57.1%	–	–
Psychiatric medications				0.7, 0.40	
Antidepressant	9, 28.1%	4, 22.2%	5, 35.7%	–	–
Mood stabilizer	2, 6.3%	2, 1.1%	0, 0%	–	–
Current psychotherapy^b^	7, 21.9%	4, 22.2%	3, 21.4%	0.003, 0.9	–
Eating disorder diagnosis				–	–
BED	–	10, 55.5%	–	–	–
OSFED-BED^c^	–	3, 16.7%	–	–	–
OSFED-Other^d^	–	5, 27.8%	–	–	–

		*M*, SD	*M*, SD	*t* or *U, p*	Cohens *d*
Age	43.5, 13.4	42.6, 12.5	44.7, 14.9	0.4, 0.67	0.15
BMI	38.6, 7.1	38.2, 7.6	39.2, 6.6	109.6, 0.53	0.14
BDI-II^e^	13.5, 11.1	18.7, 11.6	7.6, 5.2	47.5, 0.003	1.25
EDE Restraint		2.3, 1.1	0.7, 1.0	44.5, 0.001	1.52
EDE Global^f^		3.1, 0.8	1.1, 0.71	− 6.5, < 0.001	0.74
VAS- Hunger^g^		34.4, 25.9	22.1, 23.3	164.5, 0.145	0.50
VAS- Desire to eat^h^ favorite food		48.8, 27.8	29.2, 26.6	155.0, 0.065	0.72
Success at craving^i^	5.6, 2.4	7.0, 1.8	4.2, 2.6	− 2.1, 0.06	2.17
Success at resisting^i^	6.2, 2.6	6.6, 2.2	6.2, 3.1	26.0, 0.96	0.25

For continuous data, normality and outliers were determined by Kolmogorov–Smirnov and Shapiro–Wilk tests. Due to significant skew and/or kurtosis, BMI, depression symptoms, dietary restraint and visual analogue scales were compared between groups using Mann–Whitney U tests. All other variables were analyzed with independent sample t-tests for continuous data and Chi-square tests for categorical data

*BMI* body mass index, *BDI* Beck Depression Inventory-II, *EDE *Eating Disorder Examination, *M* mean, *OSFED-BED* other specified feeding or eating disorder-binge eating of limited frequency and/or duration, *OSFED-other* other specified feeding or eating disorder-other, *SD* standard deviation, *VAS* visual analogue scale, *X*^*2*^ Chi-square test statistic

^a^Group comparisons made based on dichotomous white racial group (yes/no)

^b^Participants who reported current engagement in psychotherapy (i.e., talk therapy). Engagement in therapy focused on eating or weight-related concern was exclusionary for both groups

^c^OSFED-BED diagnoses were given when the participant reported objectively large binge episodes, but of limited duration or frequency

^d^OSFED-other diagnoses were given when the participants reported subjectively large binge episodes only

^e^Equal variances were not assumed

^f^Four participants did not provide full data on the EDE, so global scores were only computed for 10 individuals

^g^Pre-task fasting rating on visual analogue scale (0 = “I am not hungry at all”; 100 = “I have never been more hungry”)

^h^Pre-task fasting rating on visual analogue scale (0 = “Not strong at all”; 100 = “Extremely strong”)

^i^Only a subset of participants completed these questions: n for BE = 9, n for control = 6

### Main effect of task condition (Crave, Resist, Watch)

The main and interactive effects of condition and group on PFC activation are presented in Table 2.

**Table 2:** Prefrontal activation during proactive inhibition task with food stimuli between adults with (*n* = 18) and without (*n* = 14) binge eating

Optode	Effect	*F*	Numerator *df*	Denominator *df*	Raw *p*-value	Partial eta-squared
1	Task	0.86	2	531.2	0.422	0.003
1	Group	0.15	1	41.8	0.705	0.003
1	Task × group	0.07	2	531.1	0.932	< 0.001
2	Task	1.22	2	479	0.295	0.005
2	Group	0.22	1	33.3	0.642	0.007
2	Task x group	0.41	2	478.9	0.665	0.002
3	Task	2.62	2	528	0.074	0.010
3	Group	1.01	1	47.3	0.320	0.021
3	Task × group	1.10	2	527.9	0.333	0.004
4	Task	0.99	2	424.3	0.372	0.005
4	Group	0.09	1	34.5	0.766	0.003
4	Task × group	0.93	2	379.1	0.395	0.005
**5**	**Task**	**4.19**	**2**	**512.8**	**0.016**	**0.016**
5	Group	1.01	1	38	0.321	0.026
5	Task × group	0.48	2	512.8	0.622	0.002
6	Task	0.87	2	399.1	0.419	0.004
6	Group	0.06	1	28.9	0.809	0.002
6	Task × group	0.64	2	399	0.526	0.003
**7**	**Task**	**6.12**	**2**	**477.3**	**0.002**	**0.025**
7	Group	3.57	1	35.6	0.067	0.091
7	Task × group	0.30	2	477.3	0.741	0.001
8	Task	1.70	2	335.1	0.184	0.010
8	Group	0.08	1	25.5	0.786	0.003
8	Task × group	0.40	2	335.1	0.674	0.002
**9**	**Task**	**3.89**	**2**	**444.2**	**0.021**	**0.017**
9	Group	3.63	1	28.1	0.067	0.114
9	Task × group	0.61	2	444.2	0.544	0.003
**10**	**Task**	**4.85**	**2**	**328.5**	**0.008**	**0.029**
10	Group	0.10	1	23.1	0.760	0.004
10	Task × group	1.23	2	328.4	0.293	0.008
**11**	**Task**	**5.02**	**2**	**490.2**	**0.007**	**0.020**
11	Group	1.06	1	33.2	0.311	0.031
11	Task × group	0.97	2	490.2	0.382	0.004
**12**	**Task**	**4.52**	**2**	**510.7**	**0.011**	**0.017**
12	Group	4.32	1	39.5	0.044	0.099
12	Task × group	2.23	2	510.7	0.109	0.009
13	Task	2.78	2	469.1	0.062	0.012
13	Group	3.93	1	32.3	0.056	0.108
13	Task × group	3.74	2	469.1	0.024	0.016

*df* = degree of freedom. Bolded values indicate significance after FDR correction. Main effects for task condition (Crave, Watch, Resist). Main effects for group (BE vs control group). Interaction of task condition by group. False detection rate Benjamini–Hochberg adjustments were applied to control for Type I Errors. Effects that were statistically significant after correcting for FDR are shown in bolded font

Across all participants (*N* = 32) there were significant small effects of task condition bilaterally in the medial superior frontal gyrus (optodes 7 and 9, $\eta_{p}^{2}$ = 0.017–0.025), dorsolateral areas (optodes 1, 5, 11, 12,13, $\eta_{p}^{2}$ = 0.010–0.029), and middle frontal gyrus (optodes 1, 5, 12; $\eta_{p}^{2}$ = 0.010–0.017). Post hoc comparisons showed greater activation during the Crave and Resist conditions compared to the Watch condition for the medial superior frontal gyrus, dorsolateral areas, and middle frontal gyrus (optodes 5, 9, 10, 11, 12; [*F* = 4.9–9.0, *p* = 0.003–0.028]). The medial superior frontal gyrus (optode 7) showed greater activation during the Resist compared to Watch condition (*F* = 12.2, *p* = 0.002).

### Main effect of group (BE vs control)

There were small to medium effects of group such that, during the task, the BE group had lesser activation than the control group bilaterally in the medial superior frontal gyrus (optodes 7 and 9; $\eta_{p}^{2}$ = 0.091–0.114), and middle frontal gyrus and dorsolateral areas (optodes 11, 12,13; $\eta_{p}^{2}$ = 0.031–0.108).

### Interaction between task condition and group

There was a small effect of the interaction between task condition and group in the middle frontal gyrus (optode 13; $\eta_{p}^{2}$ = 0.016).

## Discussion

This preliminary study utilized fNIRS to evaluate neural correlates of inhibition during a food-based reappraisal task in individuals with obesity. Contrary to hypotheses, we did not find a statistically significant link between BE status and activation of the PFC during attempted food-based reappraisal.

Our finding that compared to the Watch condition, the Crave and Resist conditions were associated with significantly greater activation bilaterally in the medial superior frontal gyrus, dorsolateral areas, and middle frontal gyrus suggest that deficits in the PFC are a potential neural basis for food-based cognitive reappraisal deficits among individuals with obesity. This is consistent with several studies that have implicated the PFC in obesity [8, 12, 16]. Importantly, the Crave and Resist conditions provided more complex instructions compared to watching the non-food videos, which may explain differential activation during these tasks, regardless of food stimuli. The lack of significant difference between the Crave and Resist conditions may suggest that a purely cognitive manipulation is not sufficient to evoke detectable differences while considering one’s response to food. The lack of behavioral components of reappraisal tasks makes it difficult to rule out this possibility and more comprehensive assessment paradigms are needed. Additionally, a comparison group with a lower BMI is needed to determine whether differential PFC activation across conditions is related to obesity.

One possible explanation for the lack of significant differences in neural activation between those with and without BE is that BE is not linked to food-based reappraisal difficulties. However, this is unlikely given that other studies have implicated hypoactivation in the PFC [4, 5, 9, 12] in the associations among BE and deficits in other components of (food and non-food based) proactive inhibition [4, 16]. Methodological limitations of this study might have diminished our ability to detect group differences across task conditions. For example, there were group differences in self-reported ability to successfully Crave food. Future studies concurrently assessing various components of food-related inhibition processing will be crucial to determine the relevance of reappraisal for BE compared to other components of inhibitory control.

### Strengths and limits

Use of a weight-matched control group is a strength of the study, as differences in inhibitory control across weight groups have been demonstrated [12]. Another strength is the use of fNIRS, which has several advantages over traditional neuroimaging techniques, including its use in real-world environments [18]. However, fNIRS does not measure activation in the anterior PFC and deeper sub-cortical areas of the limbic system, which may interact with inhibition processes. Other limitations include malfunction in three of the fNIRS optodes, and lack of data on participant medical comorbidities. Lack of data for food stimuli valence/arousal is a limitation because these qualities have been shown to elicit implicit approach biases. Greater liking for the food images compared to non-food images, in addition to inhibitory control differences, might have contributed to differences in activation across conditions. We also did not include participants across the weight strata, which could have been useful for understanding the link between neural correlates of proactive control and BMI. Finally, similar to the methodological limitations of many other studies measuring food-based inhibitory control, we did not use a task wherein food was consumed. As fNIRS can provide unique opportunities to measure neural activities while eating or exposed to food in naturalistic environments (e.g., restaurant, home), future research could measure neural correlates of cravings that precede binge-eating episodes in individuals’ daily life. Assessment of neural activation in daily life that is connected to subsequent eating behavior may improve the ecological validity of neuroimaging research on BE.

Although our findings contrast with prior literature showing differences in neural underpinnings of inhibitory control among adults with and without BE, this preliminary study adds to growing literature on the role of cognitive reappraisal in obesity/BE. The Crave–Resist task engaged activation of the medial superior frontal gyrus, dorsolateral areas, and middle frontal gyrus among adults with obesity. However, significant differences in PFC activation during food-related cognitive reappraisal between individuals with or without BE were not detected, potentially due to the limitations of the methods used to study these phenomena. Future research is needed with multimodal assessment, larger samples and longitudinal designs to further determine the precise neural signatures of BE among adults with obesity.

### What is already known on this subject?

- A large percentage of adults with obesity report binge eating.
- Individuals with obesity demonstrate deficits in food-related inhibitory control.
- Binge eating, among adults with obesity, may be underpinned by deficits in food-related inhibition processes.
- Areas of the prefrontal cortex (e.g., superior frontal gyrus, the middle frontal gyrus and inferior frontal gyrus, orbitofrontal cortex) and extended inhibitory control networks (parietal cortex, insula, cuneus, supplementary motor area) have been implicated in inhibitory control deficits among adults with high weight.

### What this study adds?

- Among individuals with obesity, there was greater activation in the medial superior frontal gyrus, dorsolateral areas, and middle frontal gyrus while attempting to crave or resist foods compared to while watching non-food videos.
- The medial superior frontal gyrus showed greater activation while attempting to resist foods compared to while watching non-food videos, among individuals with obesity.
- Among individuals with obesity, binge eating was not linked to deficits in ability to recruit the prefrontal cortex while attempting to reappraise food-cues.

## Supplementary information

**Additional file1**: **Figure S1**. fNIRS optode locations on brain surface image adapted from Ayaz et al. (2012). **Figure S2**. Schematic of the Crave–Restist task. **Table S1.** Prefrontal activation during cognitive reappraisal task with food stimuli between adults with (n = 18) and without (n = 14) binge eating, controlling for EDE-Restraint and BDI scores. **Table S2.** Prefrontal activation during proactive inhibition task with food stimuli between adults with (n = 18) and without (n = 14) binge eating: interaction between task condition (Crave, Watch, Resist) and EDE-Restraint scores. **Table S3.** Prefrontal activation during proactive inhibition task with food stimuli between adults with (n = 18) and without (n = 14) binge eating: interaction between task condition (Crave, Watch, Resist) and BDI scores. **Table S4.** Deoxygenated hemoglobin values for prefrontal activation during cognitive reappraisal task with food stimuli between adults with (n = 18) and without (n = 14) binge eating, controlling for EDE-Restraint and BDI scores.

## Data Availability

The data that support the findings of this study are available on request from the corresponding author. The data are not publicly available due to privacy or ethical restrictions.

## Abbreviations

BE: Binge eating
BMI: Body mass index
EDE: Eating disorder examination
fNIRS: Functional near-infrared spectroscopy
BDI-II: Beck depression inventory-II

## Declarations

The opinions and assertions expressed herein are those of the authors and are not to be construed as reflecting the views of the Uniformed Services University of the Health Sciences (USUHS), or the U.S. Department of Defense.
