# Supplementary material for: Prefrontal cortex activation by binge-eating status in individuals with obesity while attempting to reappraise responses to food using functional near infrared spectroscopy
Source: Eat Weight Disord. 2023 Mar 30;28(1):34. doi: 10.1007/s40519-023-01558-z (PMC10063505; doi:10.1007/s40519-023-01558-z)
Supplement: Supplementary file 1 — Additional file1: Figure S1. fNIRS optode locations on brain surface image adapted from Ayaz et al. (2012). Figure S2. Schematic of the Crave–Restist task. Table S1. Prefrontal activation during cognitive reappraisal task with food stimuli between adults with (n = 18) and without (n = 14) binge eating, controlling for EDE-Restraint and BDI scores. Table S2. Prefrontal activation during proactive inhibition task with food stimuli between adults with (n = 18) and without (n = 14) binge eating: interaction between task condition (Crave, Watch, Resist) and EDE-Restraint scores. Table S3. Prefrontal activation during proactive inhibition task with food stimuli between adults with (n = 18) and without (n = 14) binge eating: interaction between task condition (Crave, Watch, Resist) and BDI scores. Table S4. Deoxygenated hemoglobin values for prefrontal activation during cognitive reappraisal task with food stimuli between adults with (n = 18) and without (n = 14) binge eating, controlling for EDE-Restraint and BDI scores. [file 40519_2023_1558_MOESM1_ESM.docx]

**Journal:** Eating and Weight Disorders: Studies on Anorexia, Bulimia, and Obesity

**Title:** Prefrontal cortex activation by binge-eating status in individuals with obesity while attempting to reappraise responses to food using functional near infrared spectroscopy

**Running Title:** Attempted inhibition and binge eating

Megan N. Parker, MS^1,2,3,4^*

Helen Burton Murray, PhD^1,2,5,6^*

Amani D. Piers, MS^1^

Alexandra Muratore, PhD^1^

Michael R. Lowe, PhD^1^

Stephanie M. Manasse, PhD^2**^

Hasan Ayaz, PhD^1,7,8,9,10**^

Adrienne S. Juarascio, PhD^1,2**^

^1^Department of Psychology, Drexel University, Philadelphia, PA, USA

^2^The WELL Center, Drexel University, Philadelphia, PA, USA

^3^Department of Medical and Clinical Psychology, Uniformed Services University of the Health Sciences (USUHS), Bethesda, MD, USA

^4^Section on Growth and Obesity, *Eunice Kennedy Shriver* National Institute of Child Health and Human Development (NICHD), Division of Intramural Research, National Institutes of Health (NIH), Bethesda, MD, USA

^5^Department of Medicine, Massachusetts General Hospital, Boston, MA, USA

^6^Harvard Medical School, Boston, MA, USA

^7^School of Biomedical Engineering, Science and Health Systems, Drexel University, Philadelphia, PA, USA

^8^ Drexel Solutions Institute, Drexel University, Philadelphia, PA, USA

^9^Department of Family and Community Health, University of Pennsylvania, Philadelphia, PA, USA

^10^Center for Injury Research and Prevention, Children’s Hospital of Philadelphia, Philadelphia, PA, USA

*Co-first authors

**Co-senior authors

**Correspondence:** Helen Burton Murray, PhD, Division of Gastroenterology, Massachusetts General Hospital, 55 Fruit Street, Boston, MA 02114; Telephone: 617-726-8470; E-mail: [hbmurray@mgh.harvard.edu](mailto:hbmurray@mgh.harvard.edu)

**Missing data**

Twenty adults with binge eating were enrolled in the guided self-help intervention study. Of the 20, only 18 provided usable fNIRS data. For the control group, 16 individuals without BE were enrolled. However, 2 participants did not provide usable fNIRS data and were, therefore, excluded. A total of 16 adults with BE and 14 adults without BE were included in analysis


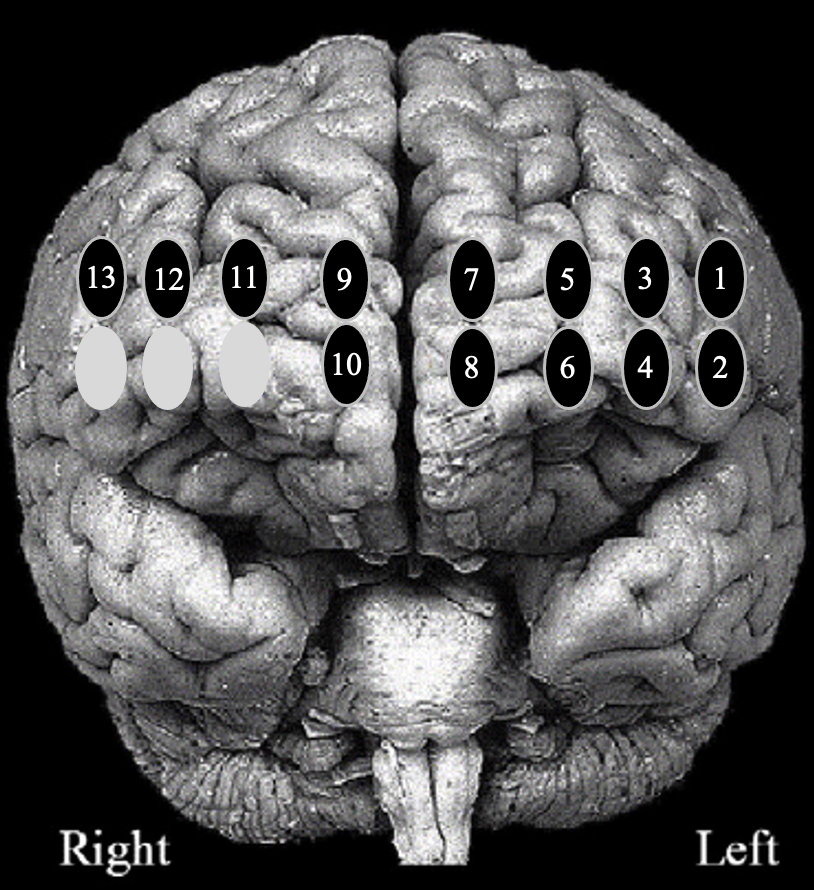
**Figure S1**. fNIRS optode locations on brain surface image adapted from Ayaz et al (2012).

Note. The 3 empty points correspond to malfunctioning optodes.

**Figure S2**. Schematic of the Crave-Restist task.

Try to resist/suppress the temptation to eat the food

Watch like you are watching a movie

500 ms

500 ms

Watch

Resist

+

+


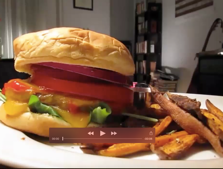

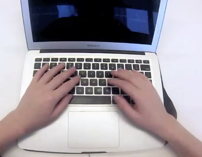


Crave

+


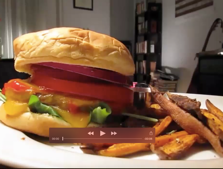


Focus on the enjoyment of consuming the food, the flavors and the sensations of eating it

5 s

Note. In the Watch block, participants were prompted to “Watch like you are watching a movie.” In the Resist block, participants were prompted to “Consider the (possible) negative consequences of (over-) consuming the food; try to resist/suppress the temptation to eat the food.” In the Crave block, were prompted to “Focus on the enjoyment of consuming the food, the flavors and the sensations of eating it.” Each video lasted 5 seconds. “Watch” videos did not contain images of food.

**Table S1.**

*Prefrontal activation during cognitive reappraisal task with food stimuli between adults with (n=18) and without (n=14) binge eating, controlling for EDE-Restraint and BDI scores*

| Optode | Effect | *F* | Numerator df | Denominator df | Raw p-value | Partial eta squared |
| --- | --- | --- | --- | --- | --- | --- |
| 1 | Task | 0.48 | 2 | 411.6 | 0.622 | 0.002 |
| 1 | Group | 0.01 | 1 | 25.0 | 0.935 | <0.001 |
| 1 | Task x Group | 0.39 | 2 | 416.1 | 0.674 | 0.002 |
| 2 | Task | 1.14 | 2 | 411.6 | 0.322 | 0.005 |
| 2 | Group | <0.01 | 1 | 20.1 | 0.949 | <0.001 |
| 2 | Task x Group | 0.85 | 2 | 423.7 | 0.429 | 0.004 |
| 3 | Task | 0.71 | 2 | 425.5 | 0.490 | 0.003 |
| 3 | Group | 0.04 | 1 | 22.8 | 0.835 | 0.002 |
| 3 | Task x Group | 0.32 | 2 | 438.6 | 0.726 | 0.001 |
| 4 | Task | 0.77 | 2 | 378.9 | 0.462 | 0.004 |
| 4 | Group | 0.01 | 1 | 16.6 | 0.911 | 0.001 |
| 4 | Task x Group | 0.93 | 2 | 379.1 | 0.395 | 0.005 |
| 5 | Task | 2.95 | 2 | 420.9 | 0.053 | 0.014 |
| 5 | Group | 0.17 | 1 | 23.9 | 0.685 | 0.007 |
| 5 | Task x Group | 0.20 | 2 | 433.2 | 0.818 | 0.001 |
| 6 | Task | 1.84 | 2 | 400.0 | 0.160 | 0.009 |
| 6 | Group | 0.02 | 1 | 16.6 | 0.886 | 0.001 |
| 6 | Task x Group | 0.31 | 2 | 399.7 | 0.734 | 0.002 |
| 7 | Task | 2.65 | 2 | 419.5 | 0.072 | 0.012 |
| 7 | Group | 0.29 | 1 | 23.2 | 0.595 | 0.012 |
| 7 | Task x Group | 0.34 | 2 | 431.1 | 0.710 | 0.002 |
| 8 | Task | 3.62 | 2 | 380.2 | 0.028 | 0.019 |
| 8 | Group | 0.04 | 1 | 18.4 | 0.836 | 0.002 |
| 8 | Task x Group | 0.81 | 2 | 379.3 | 0.447 | 0.004 |
| 9 | Task | 0.50 | 2 | 355.3 | 0.608 | 0.003 |
| 9 | Group | 1.41 | 1 | 21.3 | 0.247 | 0.062 |
| 9 | Task x Group | 0.81 | 2 | 362.8 | 0.446 | 0.004 |
| 10 | Task | 1.83 | 2 | 276.0 | 0.162 | 0.013 |
| 10 | Group | 4.40 | 1 | 11.9 | 0.058 | 0.270 |
| 10 | Task x Group | 0.80 | 2 | 275.6 | 0.450 | 0.006 |
| 11 | Task | 3.20 | 2 | 436.2 | 0.042 | 0.014 |
| 11 | Group | 1.76 | 1 | 21.7 | 0.199 | 0.075 |
| 11 | Task x Group | 1.02 | 2 | 451.5 | 0.362 | 0.004 |
| 12 | Task | 2.18 | 2 | 436.8 | 0.115 | 0.010 |
| 12 | Group | 1.14 | 1 | 23.2 | 0.296 | 0.047 |
| 12 | Task x Group | 3.34 | 2 | 450.5 | 0.036 | 0.015 |
| 13 | Task | 2.66 | 2 | 435.7 | 0.071 | 0.012 |
| 13 | Group | 3.04 | 1 | 20.5 | 0.096 | 0.129 |
| 13 | Task x Group | 4.07 | 2 | 449.6 | 0.018 | 0.018 |

Note. df = degree of freedom. Interaction of task condition by group. Main effects for task condition (Crave, Watch, Resist). Main effects for group (BE vs OBC). No significant differences after FDR correction.

**Table S2.**

*Prefrontal activation during proactive inhibition task with food stimuli between adults with (n=18) and without (n=14) binge eating: interaction between task condition (Crave, Watch, Resist) and EDE-Restraint scores*

| Optode | *F* | Numerator df | Denominator df | Raw  p-value | Partial eta squared |
| --- | --- | --- | --- | --- | --- |
| 1 | 1.08 | 2 | 448.4 | 0.340 | 0.005 |
| 2 | 1.74 | 2 | 444.8 | 0.177 | 0.008 |
| 3 | 1.02 | 2 | 456.6 | 0.361 | 0.004 |
| 4 | 0.92 | 2 | 374.5 | 0.399 | 0.005 |
| 5 | 2.11 | 2 | 451.1 | 0.122 | 0.009 |
| 6 | 1.59 | 2 | 374.8 | 0.206 | 0.008 |
| 7 | 1.57 | 2 | 451.5 | 0.210 | 0.007 |
| 8 | 1.87 | 2 | 338.2 | 0.156 | 0.011 |
| 9 | 0.92 | 2 | 390.1 | 0.397 | 0.005 |
| 10 | 2.79 | 2 | 295.5 | 0.063 | 0.019 |
| 11 | 3.72 | 2 | 464.3 | 0.025 | 0.016 |
| 12 | 3.87 | 2 | 466.8 | 0.021 | 0.016 |
| **13** | **6.33** | **2** | **458.2** | **0.002** | **0.027** |

Note. df = degree of freedom. Bolded values indicate significance after FDR correction.

**Table S3.**

*Prefrontal activation during proactive inhibition task with food stimuli between adults with (n=18) and without (n=14) binge eating: interaction between task condition (Crave, Watch, Resist) and BDI scores*

| Optode | *F* | Numerator df | Denominator df | Raw  p-value | Partial eta squared |
| --- | --- | --- | --- | --- | --- |
| 1 | 0.34 | 2 | 469.8 | 0.713 | 0.001 |
| 2 | 0.75 | 2 | 443.3 | 0.475 | 0.003 |
| 3 | 0.18 | 2 | 465.7 | 0.832 | 0.001 |
| 4 | 0.57 | 2 | 240.2 | 0.565 | 0.005 |
| 5 | 2.66 | 2 | 462.9 | 0.071 | 0.011 |
| 6 | 2.11 | 2 | 210.9 | 0.123 | 0.020 |
| 7 | 2.37 | 2 | 464.6 | 0.095 | 0.010 |
| 8 | 4.14 | 2 | 192.0 | 0.017 | 0.041 |
| 9 | 0.98 | 2 | 409.2 | 0.376 | 0.005 |
| 10 | 3.70 | 2 | 217.1 | 0.026 | 0.033 |
| 11 | 1.95 | 2 | 465.6 | 0.143 | 0.008 |
| 12 | 0.85 | 2 | 471.8 | 0.429 | 0.004 |
| 13 | 0.39 | 2 | 456.2 | 0.676 | 0.002 |

Note. df = degree of freedom. No significant differences after FDR correction.

**Table S4.**

*Deoxygenated hemoglobin values for prefrontal activation during cognitive reappraisal task with food stimuli between adults with (n=18) and without (n=14) binge eating, controlling for EDE-Restraint and BDI scores*

| Optode | Effect | *F* | Numerator df | Denominator df | Raw p-value | Partial eta squared |
| --- | --- | --- | --- | --- | --- | --- |
| 1 | Task | 2.51 | 2 | 527.9 | 0.082 | 0.009 |
| 1 | Group | 0.86 | 1 | 29.0 | 0.362 | 0.029 |
| 1 | Task x Group | 0.95 | 2 | 527.9 | 0.386 | 0.004 |
| 2 | Task | 0.35 | 2 | 456.9 | 0.703 | 0.002 |
| 2 | Group | 0.31 | 1 | 23.7 | 0.583 | 0.013 |
| 2 | Task x Group | 0.75 | 2 | 456.9 | 0.473 | 0.003 |
| 3 | Task | 4.31 | 2 | 479.2 | 0.014 | 0.018 |
| 3 | Group | 0.53 | 1 | 27.2 | 0.473 | 0.019 |
| 3 | Task x Group | 0.28 | 2 | 479.2 | 0.759 | 0.001 |
| 4 | Task | 1.03 | 2 | 418.4 | 0.356 | 0.005 |
| 4 | Group | 0.27 | 1 | 21.4 | 0.611 | 0.012 |
| 4 | Task x Group | 1.22 | 2 | 418.4 | 0.297 | 0.006 |
| 5 | Task | 2.95 | 2 | 501.1 | 0.053 | 0.012 |
| 5 | Group | 0.14 | 1 | 29.2 | 0.715 | 0.005 |
| 5 | Task x Group | 0.11 | 2 | 501.1 | 0.893 | <0.001 |
| 6 | Task | 0.11 | 2 | 333.2 | 0.900 | 0.001 |
| 6 | Group | 0.03 | 1 | 30.7 | 0.863 | 0.001 |
| 6 | Task x Group | 0.03 | 2 | 333.2 | 0.974 | <0.001 |
| 7 | Task | 2.39 | 2 | 410.3 | 0.093 | 0.012 |
| 7 | Group | 0.70 | 1 | 27.5 | 0.410 | 0.025 |
| 7 | Task x Group | 0.37 | 2 | 410.3 | 0.690 | 0.002 |
| 8 | Task | 0.39 | 2 | 299.0 | 0.676 | 0.003 |
| 8 | Group | 0.59 | 1 | 30.8 | 0.449 | 0.019 |
| 8 | Task x Group | 0.26 | 2 | 299.0 | 0.770 | 0.002 |
| 9 | Task | 2.55 | 2 | 445.6 | 0.079 | 0.011 |
| 9 | Group | 1.01 | 1 | 27.0 | 0.323 | 0.036 |
| 9 | Task x Group | 1.40 | 2 | 445.6 | 0.248 | 0.006 |
| 10 | Task | 1.18 | 2 | 312.8 | 0.308 | 0.007 |
| 10 | Group | 0.76 | 1 | 19.2 | 0.396 | 0.038 |
| 10 | Task x Group | 0.72 | 2 | 312.8 | 0.487 | 0.005 |
| 11 | Task | 2.29 | 2 | 531.8 | 0.102 | 0.009 |
| 11 | Group | 0.33 | 1 | 30.2 | 0.568 | 0.011 |
| 11 | Task x Group | 1.69 | 2 | 531.8 | 0.185 | 0.006 |
| 12 | Task | 1.39 | 2 | 528.5 | 0.251 | 0.005 |
| 12 | Group | 1.14 | 1 | 30.0 | 0.295 | 0.036 |
| 12 | Task x Group | 0.69 | 2 | 528.5 | 0.504 | 0.003 |
| 13 | Task | 2.76 | 2 | 508.6 | 0.064 | 0.011 |
| 13 | Group | 0.47 | 1 | 30.1 | 0.500 | 0.015 |
| 13 | Task x Group | 0.19 | 2 | 508.6 | 0.827 | 0.001 |

Note. df = degree of freedom. Interaction of task condition by group. Main effects for task condition (Crave, Watch, Resist). Main effects for group (BE vs OBC). No significant differences after FDR correction.
